# Dataset for solving a hybrid flexibility strategy on personnel scheduling problem in the retail industry

**DOI:** 10.1016/j.dib.2020.106066

**Published:** 2020-07-24

**Authors:** Andrés Felipe Porto, César Augusto Henao, Héctor López-Ospina, Esneyder Rafael González, Virginia I. González

**Affiliations:** a Department of Industrial Engineering, Corporación Universitaria Americana, Barranquilla, Colombia; b Universidad del Norte, Barranquilla, Colombia

**Keywords:** Personnel scheduling, Workforce flexibility, Staffing, Tour scheduling, Multiskilling, Flexible contracts, Retail services

## Abstract

This data article describes datasets from a home improvement retail store located in Santiago, Chile. The datasets have been developed to simultaneously solve a staffing and tour scheduling problem that incorporates flexible contracts and multiskilled staff. This Data in Brief article is related to the published article “*Hybrid flexibility strategy on personnel scheduling: Retail case study*” [1]. The datasets contain real, processed, and simulated data. Regarding the real and processed datasets, they are presented for three different store sizes (4, 5 or 6 departments). Real datasets include information about the employment-contract characteristics, cost parameters, and a forecast of the number of employees required in each department for each day of the week and each time period into which the operating day is divided. As regards the data processed for the case study, they include the set of skill sets considering that the employees can be trained in a maximum of two store departments. Regarding the simulated datasets, they include information about the random parameter of staff demand in each store department. The simulated data are presented in 90 text files classified by: (i) Store size (4, 5 or 6 departments). (ii) Coefficient of variation (10, 20, 30%). (iii) Instance identification number (10 instances per scenario that resulted from combining the store sizes and coefficients of variation). Researchers can use the datasets for benchmarking the performance of different approaches with the one presented by Porto et al. [1], and in consequence, they can find solutions to the same (or similar) type of personnel scheduling problem. The dataset includes an Excel workbook that can be used to randomly generate staff demand instances according to a chosen coefficient of variation.

Specifications table

**Table:** 

Subject	Management Science and Operations Research
Specific subject area	Personnel scheduling
Type of data	Tables, Figures, Text files, Excel workbook
How data were acquired	Real and processed data from a retail store and simulated data generated using a Monte Carlo simulation in an Excel workbook
Data format	Mixed (raw and analyzed)
Parameters for data collection	Real and processed datasets are presented for three different store sizes. Real data including the employment-contract characteristics, cost parameters, and a forecast of the number of employees required in each department by weekday and time period of the day. Processed data including the set of skill sets. Simulated data containing randomly generated staff demand in each store department
Description of data collection	Real and processed data were collected from a home improvement retail store. Simulated data were generated using the Excel formulas of inverse probability distribution and random values. The outputs are presented in 90 text files classified by: (i) Store size (4, 5 or 6 departments). (ii) Coefficient of variation (10, 20, 30%). (iii) Instance identification number (10 per scenario)
Data source location	Retail store in Santiago, Chile
Data accessibility	Data can be downloaded from: *Mendeley repository name*: Dataset for solving a hybrid flexibility strategy on personnel scheduling problem in the retail industry *Data identification number*: doi:10.17632/rzf6whbmg6.1 *Direct URL to data*: https://data.mendeley.com/datasets/rzf6whbmg6/draft?a=c2d30c00–5d6a-40bc-8bc5–04bcfc8329d8
Related research article	Porto, A. F., Henao, C. A., López-Ospina, H., & González, E. R. (2019). Hybrid flexibility strategy on personnel scheduling: Retail case study. *Computers & Industrial Engineering*, *133*, 220–230.

## Value of the data

• The datasets contain real, processed, and simulated information that can be used to simultaneously solve a staffing and tour scheduling problem that incorporates flexible contracts and multiskilled staff, considering a retail store with uncertain demand.
• Researchers can use the datasets for benchmarking the performance of different approaches with the one presented by Porto et al. [1], and in consequence, they can find solutions to the same (or similar) type of personnel scheduling problem.
• The data from this research can be useful to determine which are the staffing levels by contract type, the cost-effective multiskilling levels, and the weekly shift programming that minimize the costs associated with training and over/understaffing in a retail store.
• Researchers can use these data to solve other types of personnel scheduling problems, such as a shift scheduling problem, a day-off scheduling problem, or a personnel assignment problem.
• The datasets include an Excel workbook that can be used to randomly generate staff demand instances according to a chosen coefficient of variation.

## 1 Data description

The human resources management in the retail industry faces predictable phenomena such as demand seasonality, as well as unpredictable phenomena such as demand uncertainty and unscheduled staff absenteeism [1]. Such phenomena produce periods of over and understaffing that can increase labor costs and deteriorate customer service levels (CSL) [1]. To minimize this mismatch between employee supply and demand, companies have used different labor flexibility strategies to solve personnel scheduling problems [2]. Porto et al. [1] expressed that there are four type of strategies typically implemented: flexible contracts, multiskilled staff, collaborative teams and temporary employees.

The database presented in this article was used by Porto et al. [1] to simultaneously solve a staffing and tour scheduling problem that combines the following two labor flexibility strategies: (i) flexible contracts, which allow to relax the duration of the shifts and the number of weekly hours employees must work; and (ii) multiskilled staff, employees trained to work on multiple departments, which allows store managers to transfer available multiskilled employees from overstaffed departments to understaffed departments.

Staffing is a type of personnel scheduling problem that determines how many employees are required in each type of contract, and how many of them will be multiskilled employees and in which task types (or store departments). In addition, tour scheduling is another type of personnel scheduling problem where days-off and shifts are scheduled simultaneously over a given planning horizon (typically one week). The solution of a staffing and tour scheduling problem must minimize labor costs while maintaining or improving the CSL.

The data presented in the next sections are derived from a home improvement retail store in Santiago, Chile. In this store the employees are assigned to different departments, each of which constitutes a store business unit and at that level is where the store's training and employee scheduling decisions are made. This database contains real, processed, and simulated data that will be described below.

### 1.1 Real data

The real data include information about the sets and parameters that can be used to solve a hybrid flexibility strategy on personnel scheduling problem in the retail industry. Table 1 shows the notation, description, and values of these data.

**Table 1:** Real data description from a retail store with six departments.

Notation	Description	Value

*Sets*		

*D*	Days of the week, indexed by *d*.	D={1,2,3,4,5,6,7}
*P*	Time periods into which the store's operating day is divided, indexed by *p*.	P={1,2,3,4,…,28}
*C*	Contract types, indexed by *c*.	C={1,2} such that c=1→FT45; c=2→PT30
*T*	Shifts per day, indexed by *t*. Each shift is defined by a start and end time.	T={1,2,3,4,…,56}
*J*	Workday types, indexed by *j*. Defines the length of a shift.	J={1,2,3,4} such that j=1→5hrsperday; j=2→6hrsperday; j=3→9hrsperday; j=4→10hrsperday
*L*	Store departments, indexed by *l*.	L={1,2,3,4,5,6}

*Additional model sets derived from the sets defined above*		

*J_c_*	Workdays for employees with contract type *c*, ∀ *c* ∈ *C*, *J_c_*⊆*J*. Indicating that each contract type may have different feasible shift durations.	$J_{1}=\left\{3\right\};$ $J_{2}=\left\{1,2,4\right\}$
*T_cj_*	Shifts for employees with contract type *c* and workday type *j*, ∀ *c* ∈ *C, j* ∈ *J_c_*, *T_cj_*⊆ *T_c_*.	$T_{13}=\left\{3,7,11,15,19,23,27,31,35,39,42\right\};$ $T_{21}=\left\{1,5,9,13,17,21,25,29,33,37,40,43,45,47,49,51,53,55,56\right\};$ $T_{22}=\left\{2,6,10,14,18,22,26,30,34,38,41,44,46,48,50,52,54\right\};$ $T_{24}=\left\{4,8,12,16,20,24,28,32,36\right\}$

*Parameters*		

*U*	Cost per understaffed employee-period.	61 *US*$; /*period*
*O*	Cost per overstaffed employee-period.	15 *US*$; /*period*
*G_c_*	Base wage of an employee according to the contract type *c*, ∀ *c* ∈ *C*.	$G_{1}=100\;US\$;/week;$$G_{2}=90\;US\$;/week$
*r_ldp_*	Number of employees required in department *l*, on day *d*, in period *p*, ∀ *l* ∈ *L*, *d* ∈ *D*, *p* ∈ *P*.	See Table 2
*s_t_*	Start time of shift *t*, ∀ *t* ∈ *T*.	$s_{t}=8\;\forall t=1,2,3,4;$$s_{t}=8.5\;\forall t=5,6,7,8;$ $s_{t}=9\;\forall t=9,10,11,12;$$s_{t}=9.5\;\forall t=13,14,15,16;$ $s_{t}=10\;\forall t=17,18,19,20;$$s_{t}=10.5\;\forall t=21,22,23,24;$ $s_{t}=11\;\forall t=25,26,27,28;$$s_{t}=11.5\;\forall t=29,30,31,32;$ $s_{t}=12\;\forall t=33,34,35,36;$$s_{t}=12.5\;\forall t=37,38,39;$ $s_{t}=13\;\forall t=40,41,42;$$s_{t}=13.5\;\forall t=43,44;$ $s_{t}=14\;\forall t=45,46;$$s_{t}=14.5\;\forall t=47,48;$$s_{t}=15\;\forall t=49,50;$ $s_{t}=15.5\;\forall t=51,52;$$s_{t}=16\;\forall t=53,54;$$s_{t}=16.5\;\forall t=55;$ $s_{t}=17\;\forall t=56$
*e_t_*	End time of shift *t*, ∀ *t* ∈ *T*.	$e_{t}=13\;\forall t=1;$$e_{t}=13.5\;\forall t=5;$$e_{t}=14\;\forall t=2,9;$ $e_{t}=14.5\;\forall t=6,13;$$e_{t}=15\;\forall t=10,17;$$e_{t}=15.5\;\forall t=14,21;$ $e_{t}=16\;\forall t=18,25;$$e_{t}=16.5\;\forall t=22,29;$ $e_{t}=17\;\forall t=3,26,33;$$e_{t}=17.5\;\forall t=7,30,37;$ $e_{t}=18\;\forall t=4,11,34,40;$$e_{t}=18.5\;\forall t=8,15,38,43;$ $e_{t}=19\;\forall t=12,19,41,45;$$e_{t}=19.5\;\forall t=16,23,44,47;$ $e_{t}=20\;\forall t=20,27,46,49;$$e_{t}=20.5\;\forall t=24,31,48,51;$ $e_{t}=21\;\forall t=28,35,50,53;$$e_{t}=21.5\;\forall t=32,39,52,55;$ $e_{t}=22\;\forall t=36,42,54,56$
*u_p_*	Start time of period *p*, ∀ *p* ∈ *P*.	$u_{p}=\{8,8.5,9,9.5,10,10.5,11,11.5,12,12.5,13,13.5,14,14.5,15,$ 15.5, 16, 16.5, 17, 17.5, 18, 18.5, 19, 19.5, 20, 20.5, 21, 21.5}
*b_p_*	End time of period *p*, ∀ *p* ∈ *P*.	$b_{p}=\{8.5,9,9.5,10,10.5,11,11.5,12,12.5,13,13.5,14,14.5,15,15.5,$ 16, 16.5, 17, 17.5, 18, 18.5, 19, 19.5, 20, 20.5, 21, 21.5, 22}
*h_t_*	Length of shift *t*, ∀ *t* ∈ *T*.	$h_{t}=5\;\forall t\in T_{21};$$h_{t}=6\;\forall t\in T_{22};\;h_{t}=9\;\forall t\in T_{13};$ $h_{t}=10\;\forall t\in T_{24}$
*e_c_*	Number of weekly working hours according to the contract type *c*, ∀ *c* ∈ *C*.	$e_{1}=45\;hrs;$$e_{2}=30\;hrs$

Regarding the sets, it presents the days of the week that the store is operating (*D*), the time periods into which the operating day is divided (*P*), the contract types (*C*), shifts (*T*), workday types (*J*), store departments (*L*), workdays for each contract type (*J_c_*) and the shifts for each contract type and workday type (*T_cj_*). As for the parameters, it presents the costs of staff shortage (*U*) and staff surplus (*O*), and the base wage for each type of contract (*G_c_*). Finally, it provides some additional parameters which are the start and end times of shifts (*s_t_, e_t_*), the start and end times of period (*u_p_, b_p_*), the length of each shift (*h_t_*), and the number of weekly working hours according to the contract type (*e_c_*).

Particularly, the parameter associated to the forecast of the number of employees required in each department for each day of the week and period (*r_ldp_*) is presented in Table 2. This staff demand is expressed in terms of 28 time periods between 8:00 and 22:00, each period has a length of 30 min.

**Table 2:** Forecast of the number of employees required in each department for each day of the week (*D*) and each time period into which the retail store's operating day is divided (*P*).

*D*	*P*																											

**1**	**2**	**3**	**4**	**5**	**6**	**7**	**8**	**9**	**10**	**11**	**12**	**13**	**14**	**15**	**16**	**17**	**18**	**19**	**20**	**21**	**22**	**23**	**24**	**25**	**26**	**27**	**28**

*Department 1*																												

**1**	1	1	1	1	1	1	1	1	1	1	1	1	1	1	1	1	1	1	1	1	1	1	1	1	1	1	1	1
**2**	1	1	1	1	1	1	1	1	1	1	1	1	1	1	1	1	1	1	1	1	1	1	1	1	1	1	1	1
**3**	1	1	1	1	1	1	1	1	1	1	1	1	1	1	1	1	1	1	1	1	1	1	1	1	1	1	1	1
**4**	1	1	1	1	1	1	1	1	1	1	1	1	1	1	1	1	1	1	1	1	1	1	1	1	1	1	1	1
**5**	1	1	1	1	1	1	1	1	1	1	1	1	1	1	1	1	1	1	1	1	1	1	1	1	1	1	1	1
**6**	1	1	1	1	1	1	1	1	2	2	1	1	1	1	1	1	2	2	2	2	2	2	2	2	2	2	1	1
**7**	1	1	1	1	1	1	1	1	1	1	1	1	1	1	1	1	1	1	1	1	1	1	1	1	1	1	1	1

*Department 2*																												

**1**	1	1	1	1	2	2	2	2	2	2	2	2	2	2	2	2	2	2	3	3	3	3	3	3	3	3	1	1
**2**	1	1	1	1	2	2	2	2	2	2	2	2	2	2	2	2	3	3	3	3	3	3	3	3	3	3	2	2
**3**	1	1	1	1	2	2	2	2	3	3	2	2	2	2	2	2	2	2	3	3	3	3	3	3	3	3	2	2
**4**	1	1	1	1	1	1	2	2	2	2	2	2	2	2	2	2	2	2	2	2	2	2	2	2	3	3	2	2
**5**	1	1	1	1	2	2	2	2	3	3	2	2	2	2	2	2	3	3	3	3	3	3	4	4	3	3	2	2
**6**	1	1	1	1	2	2	2	2	2	2	3	3	3	3	2	2	3	3	3	3	3	3	3	3	3	3	2	2
**7**	1	1	1	1	1	1	2	2	2	2	2	2	2	2	2	2	2	2	3	3	3	3	2	2	2	2	1	1

*Department 3*																												

**1**	2	2	2	2	3	3	3	3	3	3	3	3	3	3	3	3	4	4	3	3	3	3	4	4	4	4	2	2
**2**	2	2	3	3	3	3	4	4	4	4	4	4	3	3	3	3	3	3	3	3	4	4	4	4	4	4	1	1
**3**	2	2	3	3	3	3	4	4	3	3	3	3	3	3	3	3	3	3	3	3	3	3	3	3	4	4	2	2
**4**	2	2	3	3	4	4	4	4	4	4	3	3	3	3	3	3	3	3	3	3	3	3	3	3	3	3	2	2
**5**	2	2	3	3	4	4	4	4	3	3	3	3	3	3	4	4	4	4	3	3	4	4	4	4	4	4	2	2
**6**	2	2	3	3	4	4	5	5	5	5	4	4	4	4	4	4	4	4	4	4	4	4	4	4	4	4	1	1
**7**	2	2	2	2	3	3	4	4	4	4	5	5	4	4	4	4	4	4	4	4	4	4	4	4	3	3	1	1

*Department 4*																												

**1**	1	1	2	2	2	2	3	3	3	3	3	3	3	3	3	3	3	3	3	3	3	3	4	4	4	4	2	2
**2**	2	2	2	2	3	3	3	3	3	3	3	3	3	3	3	3	3	3	3	3	4	4	4	4	4	4	2	2
**3**	2	2	2	2	2	2	3	3	3	3	3	3	3	3	3	3	3	3	4	4	3	3	3	3	4	4	2	2
**4**	2	2	2	2	3	3	3	3	3	3	3	3	3	3	3	3	3	3	3	3	4	4	4	4	4	4	2	2
**5**	1	1	2	2	3	3	3	3	3	3	3	3	3	3	3	3	3	3	3	3	4	4	4	4	4	4	2	2
**6**	2	2	2	2	3	3	3	3	4	4	4	4	4	4	4	4	4	4	5	5	5	5	5	5	4	4	2	2
**7**	1	1	2	2	2	2	3	3	5	5	6	6	5	5	4	4	6	6	7	7	6	6	6	6	4	4	1	1

*Department 5*																												

**1**	1	1	1	1	1	1	1	1	1	1	1	1	1	1	1	1	1	1	1	1	1	1	1	1	1	1	1	1
**2**	1	1	1	1	1	1	1	1	1	1	1	1	1	1	1	1	1	1	1	1	1	1	1	1	1	1	1	1
**3**	1	1	1	1	1	1	1	1	1	1	1	1	1	1	1	1	1	1	1	1	1	1	1	1	1	1	1	1
**4**	1	1	1	1	1	1	1	1	1	1	1	1	1	1	1	1	1	1	1	1	1	1	1	1	1	1	1	1
**5**	1	1	1	1	1	1	1	1	1	1	1	1	1	1	1	1	1	1	1	1	1	1	1	1	1	1	1	1
**6**	1	1	1	1	1	1	1	1	2	2	1	1	1	1	1	1	2	2	2	2	2	2	2	2	1	1	1	1
**7**	1	1	1	1	1	1	1	1	2	2	2	2	2	2	2	2	2	2	2	2	2	2	2	2	2	2	1	1

*Department 6*																												

**1**	1	1	1	1	1	1	2	2	2	2	2	2	2	2	2	2	2	2	2	2	2	2	2	2	2	2	1	1
**2**	1	1	1	1	1	1	2	2	2	2	2	2	2	2	2	2	2	2	2	2	3	3	3	3	3	3	2	2
**3**	1	1	1	1	2	2	2	2	2	2	2	2	2	2	2	2	2	2	3	3	3	3	3	3	3	3	1	1
**4**	1	1	1	1	1	1	2	2	1	1	2	2	2	2	2	2	2	2	2	2	2	2	2	2	2	2	2	2
**5**	1	1	1	1	1	1	1	1	1	1	1	1	1	1	1	1	1	1	1	1	1	1	1	1	1	1	1	1
**6**	1	1	1	1	1	1	2	2	2	2	3	3	2	2	2	2	3	3	3	3	3	3	3	3	3	3	2	2
**7**	1	1	1	1	1	1	2	2	2	2	3	3	3	3	2	2	3	3	4	4	4	4	4	4	3	3	1	1

In order to maintain the optimization problem computationally tractable, Porto et al. [1] considered three store size (SS). Each SS represents the number of departments that the store has. The first SS has four departments, the second SS included five departments, and the third SS included six departments. Tables 1 and 2 show a SS of six departments, but to represent the other SS it would only be necessary to adjust *L* to the number of departments (i.e., L={1,2,3,4} for the SS of four departments and L={1,2,3,4,5} for the SS of 5 departments). Also, for the parameter *r_ldp_*, the demand for the corresponding SS can be selected in the order presented in Table 2.

The retailer data associated to the case study were provided by SHIFT SpA [3], a firm that optimizes the shift schedules of thousands of employees across Latin America. Two types of contracts were defined for the addressed personnel scheduling problem, based on established practices in the Chilean retail sector. The first contract type is FT45, for full-time employees working 45 weekly hours, while the second contract type is PT30, for part-time employees working 30 weekly hours. As mentioned before, the set *C* represents the contract types, indexed by *c*, and the values c=1 indicates a FT45 contract and c=2 a PT30 contract.

As regards the workday types, there are three possible workdays in the PT30 contract (i.e., 5, 6 and 10 h) and there is one possible workday in the FT45 contract (i.e., 9 h). As noted above, the set *J* represents the workday types, indexed by *j*, and the values j=1 indicate a workday of 5 h, j=2 a workday of 6 h, j=3 a workday of 9 h and j=4 a workday of 10 h.

As a result, Table 3 shows the set of shifts per day (*T*, indexed by *t*) that was obtained for each combination of contract type and workday type (i.e. workday groups). Column 1 shows the workday groups, which are structures formed by the type of contract, the working days per week, and the working hours per day. For example, the workday group FT45 {5  ×  9} represents an employee under a full-time contract that works 45 h per week, spread over 5 days per week and 9 h per day.

**Table 3:** Shifts alternatives per working day for two contract types.

Workday groups (*days* × *h*/*day*)	Start and end time of shifts (*s_t_*-*e_t_*)			Shifts for employees with contract type *c* and workday type *j* (*T_cj_*)			N° Shifts per day |*T*|

FT45 {5 × 9}	8:00–17:00	8:30–17:30	9:00–18:00	$T_{13}=$3	7	11	11
	9:30–18:30	10:00–19:00	10:30–19:30	15	19	23	
	11:00–20:00	11:30–20:30	12:00–21:00	27	31	35	
	12:30–21:30	13:00–22:00		39	42		

PT30 {6 × 5}	8:00–13:00	8:30–13:30	9:00–14:00	$T_{21}=$1	5	9	19
	9:30–14:30	10:00–15:00	10:30–15:30	13	17	21	
	11:00–16:00	11:30–16:30	12:00–17:00	25	29	33	
	12:30–17:30	13:00–18:00	13:30–18:30	37	40	43	
	14:00–19:00	14:30–19:30	15:00–20:00	45	47	49	
	15:30–20:30	16:00–21:00	16:30–21:30	51	53	55	
	17:00–22:00			56			

PT30 {5 × 6}	8:00–14:00	8:30–14:30	9:00–15:00	$T_{22}=$2	6	10	17
	9:30–15:30	10:00–16:00	10:30–16:30	14	18	22	
	11:00–17:00	11:30–17:30	12:00–18:00	26	30	34	
	12:30–18:30	13:00–19:00	13:30–19:30	38	41	44	
	14:00–20:00	14:30–20:30	15:00–21:00	46	48	50	
	15:30–21:30	16:00–22:00		52	54		

PT30 {3 × 10}	8:00–18:00	8:30–18:30	9:00–19:00	$T_{24}=$4	8	12	9
	9:30–19:30	10:00–20:00	10:30–20:30	16	20	24	
	11:00–21:00	11:30–21:30	12:00–22:00	28	32	36	

**Total**							**56**

Columns 2 and 3 in Table 3 present detailed information for each shift belonging to a workday group. Column 2 indicates the start (*s_t_*) and end (*e_t_*) time associated to each shift *t*. Note that, the shifts can only start every 30 min and are limited by the store operating hours (i.e., 8:00–22:00). Column 3 shows for each shift presented in the Column 2 what is their respective value of *t* ∈ *T_cj_*. Note that each workday group is equivalent to one of the sets *T_cj_*, which represents the shifts for employees with contract type *c* and workday type *j*, ∀ *c* ∈ *C, j* ∈ *J_c_*, *T_cj_*⊆ *T_c_*. The workday group FT45 {5  ×  9} is equivalent to the set *T*_13_, the workday group PT30 {6  ×  5} is equivalent to the set *T*_21_, the workday group PT30 {5  ×  6} is equivalent to the set *T*_22_, and the workday group PT30 {3  ×  10} is equivalent to the set *T*_24_. The last column presents the number of shifts that can be considered for each workday group, whose total sum is equivalent to |T|=56.

Finally, a detailed description of how we estimate the personnel demand and the staff costs will be addressed in the experimental design, materials, and methods section.

### 1.2 Data processed for the case study

In this section, we present the data processed for the case study solved in Porto et al. [1], which refers to the set of skill sets (i.e., $W,\;L_{w},\;W_{l},\;W_{l}^{out}$,$W_{l}^{in}$). Table 4 shows the set of skill sets for the SS of six departments, but the building process is the same for the SS of four departments and the SS of five departments. In fact, the detailed building process of *W* for any store size will be addressed in the experimental design, materials, and methods section. The other sets are derived from *W*.

**Table 4:** Description of the data processed for the case study of a retail store with six departments.

Notation	Description	Value

*Sets*		
*W*	Set of skill sets, indexed by *w*. Includes subsets with only one skill (i.e., single-skill) and subsets with maximum two skills (i.e., multi-skill).	W={1,2,3,4,…,36}

*Additional model sets derived from the sets defined above*		

*L_w_*	Subset of store departments that are included in the skill set *w*, ∀*w* ∈ *W*, *L_w_*⊆*L*. Each subset has maximum two departments.	$L_{1}=\left\{1\right\};\;L_{2}=\left\{1,2\right\};\;L_{3}=\left\{1,3\right\};\;L_{4}=\left\{1,4\right\};$ $L_{5}=\left\{1,5\right\};\;L_{6}=\left\{1,6\right\};\;L_{7}=\left\{2,1\right\};\;L_{8}=\left\{2\right\};$ $L_{9}=\left\{2,3\right\};\;L_{10}=\left\{2,4\right\};\;L_{11}=\left\{2,5\right\};\;L_{12}=\left\{2,6\right\};$ $L_{13}=\left\{3,1\right\};\;L_{14}=\left\{3,2\right\};\;L_{15}=\left\{3\right\};\;L_{16}=\left\{3,4\right\};$ $L_{17}=\left\{3,5\right\};\;L_{18}=\left\{3,6\right\};\;L_{19}=\left\{4,1\right\};\;L_{20}=\left\{4,2\right\};$ $L_{21}=\left\{4,3\right\};\;L_{22}=\left\{4\right\};\;L_{23}=\left\{4,5\right\};\;L_{24}=\left\{4,6\right\};$ $L_{25}=\left\{5,1\right\};\;L_{26}=\left\{5,2\right\};\;L_{27}=\left\{5,3\right\};\;L_{28}=\left\{5,4\right\};$ $L_{29}=\left\{5\right\};\;L_{30}=\left\{5,6\right\};\;L_{31}=\left\{6,1\right\};\;L_{32}=\left\{6,2\right\};$ $L_{33}=\left\{6,3\right\};\;L_{34}=\left\{6,4\right\};\;L_{35}=\left\{6,5\right\};\;L_{36}=\left\{6\right\}$
*W_l_*	Skill sets that allow an employee to work in the department *l*, ∀*l* ∈ *L*, *W_l_*⊆*W*. Each skill set has 2|L|−1 skills.	$W_{1}=\left\{1,2,3,4,5,6,7,13,19,25,31\right\};$ $W_{2}=\left\{2,7,8,9,10,11,12,14,20,26,32\right\};$ $W_{3}=\left\{3,9,13,14,15,16,17,18,21,27,33\right\};$ $W_{4}=\left\{4,10,16,19,20,21,22,23,24,28,34\right\};$ $W_{5}=\left\{5,11,17,23,25,26,27,28,29,30,35\right\};$ $W_{6}=\left\{6,12,18,24,30,31,32,33,34,35,36\right\}$
$W_{l}^{out}$	Multi-skill sets where department *l* is considered the primary department of the employee, but he/she also has an additional skill to work in a secondary department, ∀*l* ∈ *L*, *W_l_*⊆*W*. Each multi-skill set has |L−1| skills.	$W_{1}^{out}=\left\{2,3,4,5,6\right\};$ $W_{2}^{out}=\left\{7,9,10,11,12\right\};$ $W_{3}^{out}=\left\{13,14,16,17,18\right\};$ $W_{4}^{out}=\left\{19,20,21,23,24\right\};$ $W_{5}^{out}=\left\{25,26,27,28,30\right\};$ $W_{6}^{out}=\left\{31,32,33,34,35\right\}$
$W_{l}^{in}$	Multi-skill sets where an employee has a primary department different to *l*, but he/she also has an additional skill to work in the department *l*, ∀*l* ∈ *L*, *W_l_*⊆*W*. Each multi-skill set has |L−1|skills.	$W_{1}^{in}=\left\{7,13,19,25,31\right\};$ $W_{2}^{in}=\left\{2,14,20,26,32\right\};$ $W_{3}^{in}=\left\{3,9,21,27,33\right\};$ $W_{4}^{in}=\left\{4,10,16,28,34\right\};$ $W_{5}^{in}=\left\{5,11,17,23,35\right\};$ $W_{6}^{in}=\left\{6,12,18,24,30\right\}$

*Parameters*		

*M_w_*	Cost of training an employee according to the skill set *w*, ∀*w* ∈ *W*.	$M_{w}=1\;US\$;$-*week*/*multi*-*skilled* *employee* → $M_{w}=0\;\forall w=1,8,15,22,29,36;$ $M_{w}=1\;\forall w=2,3,4,5,6,7,9,10,11,12,13,14,16,17,18,19,$ 20, 21, 23, 24, 25, 26, 27, 28, 30, 31, 32, 33, 34, 35

The first set is *W* which represents the skill sets, indexed by *w*. Given that the employees can be trained in a maximum of two store departments, the cardinality of *W* is determined by the square of the SS. For example, if the SS is six departments then |W|=36, such that this set includes 6 subsets with only one skill (i.e., single-skill) and 30 subsets with maximum two skills (i.e., multi-skill). Note that, each skill set *w* is the result of the combination of each store department with itself and with the other departments. On one side, when a department is combined with itself the result is a single-skill subset. On the other hand, when a department is combined with another department the result is a multi-skill subset.

The second set is *L_w_* which represents the subset of store departments that are included in the skill set *w*, ∀*w* ∈ *W*, *L_w_*⊆*L*. According to the value of *w, L_w_* can have one or maximum two departments. On one side, if *L_w_* has a single department, it is associated with a single-skill subset. On the other hand, if *L_w_* has two departments, it is associated with a multi-skill subset. In this last case, a distinction is made between which is the primary department and which is the secondary one.

In Table 4 for example, $L_{1}=\left\{1\right\}$ is associated with the single-skill subset w=1, where employees are trained to work only in department 1. $L_{2}=\left\{1,2\right\}$ is associated with the multi-skill subset w=2, where employees are trained to work in department 1 as primary, and in department 2 as secondary. It should be noted that even though $L_{2}=\left\{1,2\right\}$ and $L_{7}=\left\{2,1\right\}$ are associated with multi-skill subsets where employees are trained to work in department 1 and department 2, they differ because their primary department is 1 and 2 respectively. This property also applies to the rest of the multi-skill subsets.

The third set is *W_l_* which represents the skill sets that allow an employee to work in the department *l*, ∀*l* ∈ *L*, *W_l_*⊆*W*. To differentiate the primary department from the secondary department, we defined $W_{l}^{out}$ and $W_{l}^{in}$. First, $W_{l}^{out}$ represents the multi-skill sets where department *l* is considered the primary department of the employee, but he/she also has an additional skill to work in a secondary department, ∀*l* ∈ *L*, *W_l_*⊆*W*. Second, $W_{l}^{in}$ represents the multi-skill sets where an employee has a primary department different to *l*, but he/she also has an additional skill to work in the department *l*, ∀*l* ∈ *L*, *W_l_*⊆*W*.

In addition, we assume a minimal training cost per each hired multiskilled employee, such that *M_w_* = 1 *US*$; -*week*/*multi*-*skilled* *employee*. Note that, the training cost is zero for the skill sets *w* with only one skill (i.e., single-skill).

Finally, we provided three files with the sets and parameters of the real and processed data written in AMPL, for each SS. The files named ‘SS4-real-processed-data.dat’, ‘SS5-real-processed-data.dat’ and ‘SS6-real-processed-data.dat’ contain the values that correspond to a SS of four departments, five departments and six departments, respectively. All files can be downloaded from the Mendeley repository that was provided in Data accessibility Section (see Specifications Table).

### 1.3 Simulated data

The forecast values of the parameter *r_ldp_*, which represents the number of employees required in department *l*, on day *d*, in period *p*, ∀ *l* ∈ *L*, *d* ∈ *D*, *p* ∈ *P*, were presented in Table 2. But also, in order to evaluate the potential benefits of the joint use of flexible contracts and multiskilled employees in the face of different changes in staffing demand, Porto et al. [1] created simulated demands considering three different variability levels in each department: CV = 10%, 20% and 30%. Such that, CV is the coefficient of variation of demand from the forecast values presented in Table 2.

Combining the SS (4, 5 and 6 departments) and the CV (10%, 20%, 30%) the resulting number of scenarios is 9. A Monte Carlo simulation was used to randomly generate instances for demand in each store department following a zero-truncated normal probability distribution (this prevents negative demand values). In our case, 10 demand instances were generated for each of the scenarios, resulting in 90 instances. These 90 simulated demands are provided in the text files listed in Table 5 and can be downloaded from the Mendeley repository that was provided in Data accessibility Section (see Specifications Table).

**Table 5:** Simulated datasets for the random realizations of the number of employees required in each department, day, and time period.

	Store size (SS)		
CV	4 Departments	5 Departments	6 Departments

10%	SS4-CV10–01.dat, SS4-CV10–02.dat, SS4-CV10–03.dat, SS4-CV10–04.dat, SS4-CV10–05.dat, SS4-CV10–06.dat, SS4-CV10–07.dat, SS4-CV10–08.dat, SS4-CV10–09.dat, SS4-CV10–10.dat	SS5-CV10–01.dat, SS5-CV10–02.dat, SS5-CV10–03.dat, SS5-CV10–04.dat, SS5-CV10–05.dat, SS5-CV10–06.dat, SS5-CV10–07.dat, SS5-CV10–08.dat, SS5-CV10–09.dat, SS5-CV10–10.dat	SS6-CV10–01.dat, SS6-CV10–02.dat, SS6-CV10–03.dat, SS6-CV10–04.dat, SS6-CV10–05.dat, SS6-CV10–06.dat, SS6-CV10–07.dat, SS6-CV10–08.dat, SS6-CV10–09.dat, SS6-CV10–10.dat

20%	SS4-CV20–01.dat, SS4-CV20–02.dat, SS4-CV20–03.dat, SS4-CV20–04.dat, SS4-CV20–05.dat, SS4-CV20–06.dat, SS4-CV20–07.dat, SS4-CV20–08.dat, SS4-CV20–09.dat, SS4-CV20–10.dat	SS5-CV20–01.dat, SS5-CV20–02.dat, SS5-CV20–03.dat, SS5-CV20–04.dat, SS5-CV20–05.dat, SS5-CV20–06.dat, SS5-CV20–07.dat, SS5-CV20–08.dat, SS5-CV20–09.dat, SS5-CV20–10.dat	SS6-CV20–01.dat, SS6-CV20–02.dat, SS6-CV20–03.dat, SS6-CV20–04.dat, SS6-CV20–05.dat, SS6-CV20–06.dat, SS6-CV20–07.dat, SS6-CV20–08.dat, SS6-CV20–09.dat, SS6-CV20–10.dat

30%	SS4-CV30–01.dat, SS4-CV30–02.dat, SS4-CV30–03.dat, SS4-CV30–04.dat, SS4-CV30–05.dat, SS4-CV30–06.dat, SS4-CV30–07.dat, SS4-CV30–08.dat, SS4-CV30–09.dat, SS4-CV30–10.dat	SS5-CV30–01.dat, SS5-CV30–02.dat, SS5-CV30–03.dat, SS5-CV30–04.dat, SS5-CV30–05.dat, SS5-CV30–06.dat, SS5-CV30–07.dat, SS5-CV30–08.dat, SS5-CV30–09.dat, SS5-CV30–10.dat	SS6-CV30–01.dat, SS6-CV30–02.dat, SS6-CV30–03.dat, SS6-CV30–04.dat, SS6-CV30–05.dat, SS6-CV30–06.dat, SS6-CV30–07.dat, SS6-CV30–08.dat, SS6-CV30–09.dat, SS6-CV30–10.dat

Each file contains the number of employees required in each store department, such that each row represents one of the 7 days of the week, and each column represents one of the 28 time periods into which the retail store's operating day is divided. The file names are identified by a three-character code *i-j-k*, where *i* = SS4, SS5, SS6 indicates the store size (4, 5 or 6 departments); *j* = CV10, CV20, CV30 indicates the coefficient of variation (CV = 10, 20 or 30%); and *k* = 01, 02, 03, 04, 05, 06, 07, 08, 09, 10 represents the instance identification number (10 instances per scenario).

**Fig. 1:**
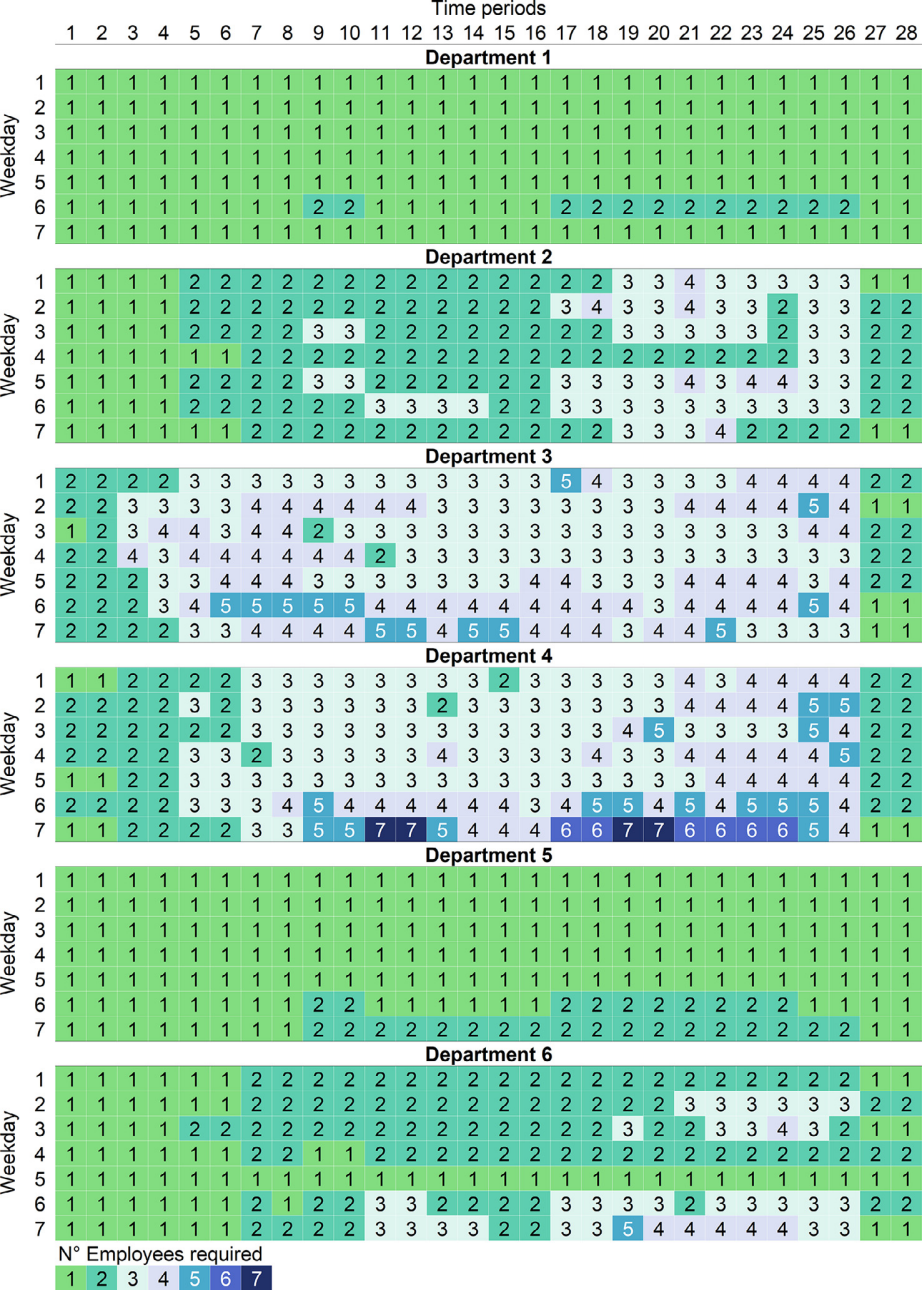
Data visualization for the first instance of the simulated demand, for the scenario with SS=6 and CV=10%.

**Fig. 2:**
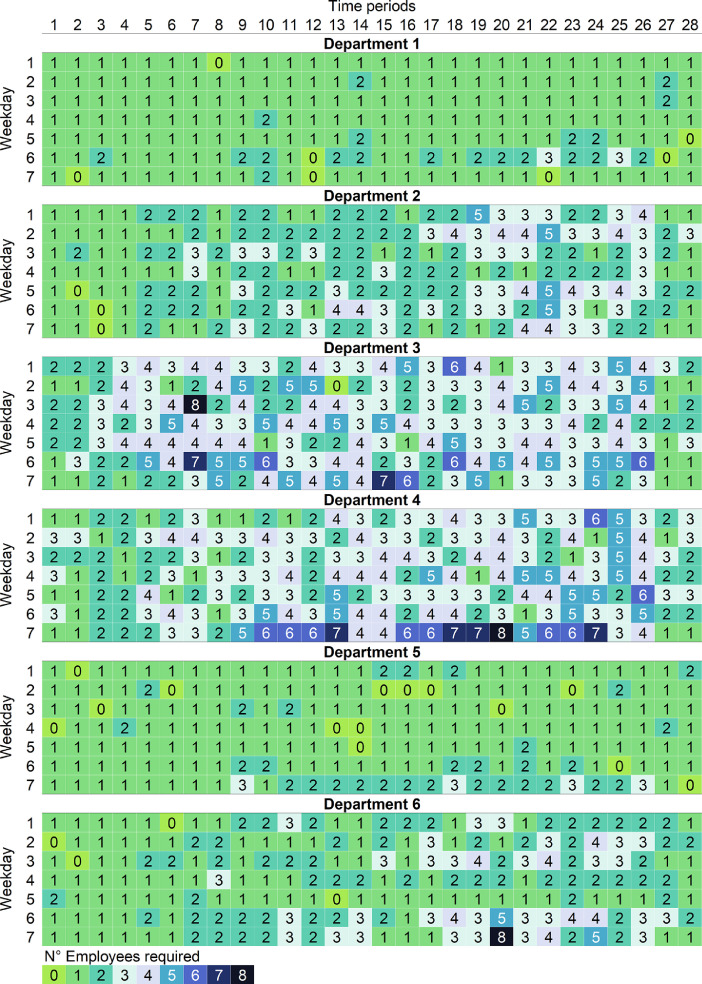
Data visualization for the first instance of the simulated demand, for the scenario with SS=6 and CV=30%.

As an example, Figs. 1 and 2 were created to visualize the simulated demands. Fig. 1 shows the first instance of the simulated demand, for the scenario with six store departments and a coefficient of variation equal to 10% (i.e., the data from the ‘SS6-CV10–01.dat’ file). And Fig. 2 shows the first instance of the simulated demand, for the scenario with six store departments and a coefficient of variation equal to 30% (i.e., the data from the ‘SS6-CV30–01.dat’ file). In both instances, departments 3 and 4 are the ones with the highest demands, and department 1 has the lowest demand, just like the forecast values of *r_ldp_* presented in Table 2. For CV=10%, the number of required employees ranges from 1 to 7, considering all departments, days, and time periods. Meanwhile for CV=30% the number of required employees ranges from 0 to 8, considering all departments, days, and time periods.

Finally, a detailed description of the tool we used to perform the Monte Carlo simulations will be addressed in the experimental design, materials, and methods section.

## 2 Experimental design, materials, and methods

In this section, we present a complete description of the experimental design and methods used to acquire the data regarding the skill sets, the staff costs, and the forecast and simulated demand. First, we describe how the skill sets were designed. Second, we explain the reasoning behind the estimation of the base wage per type of contract and the costs of training, staff shortage and staff surplus. Third, we indicate the methods used to obtain the forecast staff demand in each store department. Finally, we provide a description of the program used for the Monte Carlo simulation, which generates random parameters of staff demand.

### 2.1 Skill sets

**Fig. 3:**
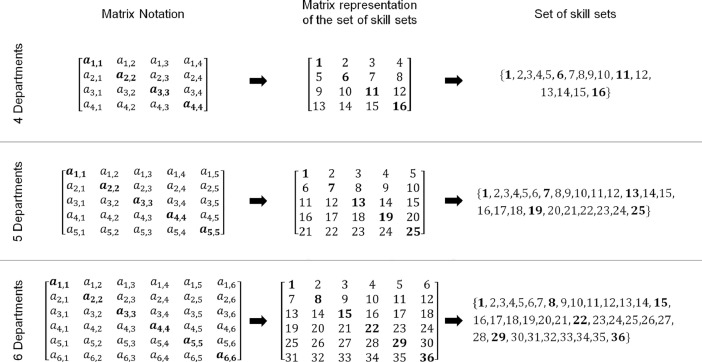
Building process of the set *W* associated with each SS.

The sets *W* were designed using square matrices such that the number of rows and columns is equal to the store size (SS). Fig. 3 shows the matrix notation, matrix representation, and linear vector for each set *W* associated with each SS (4, 5 and 6 departments). In the matrix representation of sets *W* the main diagonal represents the single-skill subsets (indicated in bold). Furthermore, the entries above and below the main diagonal represent the multi-skill subsets. Note that, in the model, the set *W* was finally written as a linear vector, thus the matrix representation is for illustration purposes only.

For example, when SS=6, the matrix notation *a*_1, 1_ is associated with the single-skill subset w=1, where employees are trained to work only in department 1. In addition, the matrix notation *a*_1, 2_ is associated with the multi-skill subset w=2, where employees are trained to work in department 1 as primary, and in department 2 as secondary. As well as that, the matrix notation *a*_2, 1_ is associated with the multi-skill subset w=7, where employees are trained to work in department 2 as primary, and in department 1 as secondary. Finally, as already explained in Section 1.2, the other skill sets (i.e.,$\;L_{w},\;W_{l},\;W_{l}^{out}$,$W_{l}^{in}$) are derived from *W*.

### 2.2 Staff costs

Regarding the cost structure, we set the base wage per type of contract according to the Chilean Labor Law effective in 2018. The weekly base wage for a full-time employee (i.e., FT45 contract) is equal to $G_{1}=100\;US\$;/week$, whereas for a part-time employee (i.e., PT30 contract) is equal to $G_{2}=90\;US\$;/week$. Note that, the weekly cost of a full-time employee is greater than the weekly cost of a part-time employee, since the former works 15-h more than the latter. However, one hour of work by a part-time employee is typically more costly than one hour of work by a full-time employee.

In regards to the cost of training associated with multi-skilled employees, we assume a minimal training cost of $M_{w}=1\;US\$;$*-week*/*multi*-*skilled* *employee*. Henao et al. [4], Henao et al. [5], Henao et al. [6], and Porto et al. [1] explain that this assumption allows for interpreting the results as an upper bound on the potential contribution of multiskilling to store performance.

As for the shortage and surplus costs, we assumed to be the same for all departments, time periods and days. The shortage cost is equivalent to the cost of the expected lost sales, and according to historical data of the retail store is 61 US$/period on average. The surplus cost represents an opportunity cost, incurred by paying for idle staff who could be assigned to other productive tasks in the store, and the average cost was determined to be 15 US$/period using historical data.

### 2.3 Demand forecast from a retail store

SHIFT SpA is a specialized firm that provides advisory services in workforce management for different companies in Latin America [3]. As mentioned before, SHIFT SpA provided the real data for our case study, which are derived of a home improvement retail store located in Santiago, Chile. In particular, the staff demand by department was obtained through a specialized software that runs in two steps: (1) forecast the sales and transactions and (2) generate the staff requirements.

In the first step, the software uses a multiple linear regression to forecast the amount of sales and number of transactions of the store by department, day, and time period. This procedure is based on historical data. To improve the estimation of the regression, the store must have at least 2–6 years of data. In the second step, the software transforms the predicted sales and transactions in a staff demand expressed in man-hours, considering the typical customer service times. Then, given a pre-established level of service, the number of employees required in each store department per day and time period is determined.

### 2.4 Monte Carlo simulation

In the Mendeley repository that was provided in Data accessibility Section (see Specifications Table), we supply an Excel workbook named ‘Monte Carlo Simulator.xlsx’ which contains the Monte Carlo simulation used to generate the instances for demand in each store department following a zero-truncated normal probability distribution (simulated data). This workbook contains one worksheet that can be used to generate demand instances one by one, for a given store department.

Since the staff demand follows a normal distribution, two parameters are required to generate the instances: (i) the forecast of the number of employees required in the department for each day of the week and each time period, which is given in Table 2; and (ii) the coefficient of variation, which can be selected by the user to represent the uncertainty in the staff demand that best fits its case study. In the Excel worksheet, these parameters or inputs are placed in a yellow fill cell, this color indicates that the values can be edited.

The rest of the input data is calculated using Excel formulas, and are presented in gray text to indicate that these cells should not be edited. In the worksheet, the standard deviation is calculated as the product between the forecast demand and the CV. Then some statistics are calculated to truncate the outputs. Since the distribution is zero-truncated, the standard score (z) and its respective quantiles were calculated.

Finally, the demand instance is calculated using the Excel formulas of inverse probability distribution and random values. The random values range between the quantile (associated with the standard value of demand equal to zero) and 1, and a step size of 0.0001. This ensures that the generated demand values are not negative, that is, that they are greater than or equal to zero. The random parameters of staff demand outputs are organized in 7 rows representing the days of the week, and 28 columns representing the time periods into which the retail store's operating day is divided. Such values are presented in blue text to indicate that these cells are the outputs and should not be edited.

## Declaration of Competing Interest

The authors declare that they have no known competing financial interests or personal relationships which have, or could be perceived to have, influenced the work reported in this article.

## Appendix A Supplementary materials

Supplementary Data S1 Supplementary Raw Research Data. This is open data under the CC BY license http://creativecommons.org/licenses/by/4.0/ Supplementary Data S1
